# The Role of T Lymphocytes in Skeletal Muscle Repair From Traumatic and Contraction-Induced Injury

**DOI:** 10.3389/fphys.2018.00768

**Published:** 2018-06-20

**Authors:** Michael R. Deyhle, Robert D. Hyldahl

**Affiliations:** Department of Exercise Sciences, Brigham Young University, Provo, UT, United States

**Keywords:** T-cell, inflammation, lymphoid, muscle damage, muscle regeneration, satellite cell, Treg, exercise

## Abstract

Skeletal muscle is prone to damage from a range of stimuli, and initiates a robust repair process that requires the participation of immune cells. Among the more well characterized immune cells involved in muscle repair are those of the myeloid lineage, including neutrophils, macrophages, monocytes, and eosinophils. More recently, studies have begun to elucidate the role of the lymphoid-derived immune cells, most notably T lymphocytes (T-cells), in the complex processes of muscle repair. Though T-cells have been traditionally been associated with pathological degeneration of skeletal muscle in disease, recent studies show that T-cells are instrumental in the repair/regeneration process following severe muscle damage in mice. Furthermore, a few studies using basic immunohistochemical assays have shown that T-cells accumulate in human skeletal muscle in the days following contraction-induced muscle damage. The functional significance of T-cells in the repair and adaptation process following contraction-induce muscle damage remains uncertain, and is an active area of intense investigation. This mini-review summarizes recent findings on the involvement of T-cells in skeletal muscle repair.

## Introduction

Both skeletal muscle injury and degenerative disease (i.e., muscular dystrophy, sarcopenia) are major public health burdens with few bona fide therapeutic interventional strategies. The extent to which future interventions can be successful at restoring muscle functional capacity in the face of injury or disease may lie in their potential to improve muscle regenerative outcomes. Apropos of this notion, recent insights have been achieved concerning the importance of skeletal muscle/immune cell interaction for effective repair outcomes, particularly as it relates to T lymphocytes (T-cells). The contributing role of T-cells in muscle regeneration as part of the whole immune response to muscle injury was recently reviewed elsewhere (Tidball, 2017). Another recent review focused on the role of a specific T-cell subset in the muscle regeneration process (Schiaffino et al., 2016). The intention of this mini-review is to summarize the current understanding of T-cells in skeletal muscle repair and regeneration following damage and the potential role of T-cells in muscle repair and adaptation following contraction-induced muscle damage.

## T-Cells in Muscle Health and Disease

T-cells have long been identified as active participants in various muscle diseases. CD8 T-cells were found to greatly increase muscle fiber necrosis in dystrophic muscle by perforin-mediated cytotoxicity (Spencer et al., 1997). Dystrophic features of dysphyrlin-deficient muscle were greatly reduced with T-cell depletion (Farini et al., 2012). T-cells are implicated in muscle cytotoxicity in idiopathic inflammatory myopathy conditions (Johnson et al., 1972; De Paepe et al., 2005; Choi et al., 2009; Graca and Kouyoumdjian, 2015; Huang et al., 2015; Limongi, 2015). Recently, T-cells were found to accumulate in the muscle of high fat-fed mice, and were instrumental in the development of insulin insensitivity (Khan et al., 2014, 2015).

With the large body of evidence connecting T-cells to muscle disease, it is not surprising that they were suspected to be of pathological significance in the first reports of T-cells infiltrating non-diseased muscle following injury (Orimo et al., 1991; McLennan, 1996). Yet, as more information regarding the role of T-cells in muscle repair from injury has emerged in recent years, a different story has unfolded. Recent studies show that conventional CD8 and CD4 T-cells, as well as regulatory T-cells (Tregs) play important roles in muscle regeneration. Several elaborate studies have demonstrated this quite clearly. For example, the loss or gain of CD8 (Zhang et al., 2014; Fu et al., 2015) or CD4 (Fu et al., 2015) T-cells robs and rescues muscle regeneration capacity, respectively. Similarly, the loss of Tregs impairs muscle repair and regeneration (Burzyn et al., 2013; Kuswanto et al., 2016).

The reason why T-cells can contribute to the pathology of several muscle diseases, and yet are essential for proper muscle regeneration in otherwise healthy muscle following injury, has not been specifically investigated. It may be that conventional T-cells are detrimental in conditions that are characterized by persistent inflammation or persistent muscle damage. Mice lacking Casitas B-lineage lymphoma-b suffer from impaired muscle regeneration that is rescued by blunting muscle CD8 T-cell infiltration (Kohno et al., 2011). However, these mice suffer from exaggerated inflammation and prolonged T-cell presence within the muscle following injury (>14 days). Consistently, studies show that in animals that display successful muscle regeneration, muscle T-cells return to pre-injury levels within 7–10 days (Burzyn et al., 2013; Zhang et al., 2014; Castiglioni et al., 2015; Fu et al., 2015). Thus, the muscle regeneration detriment owed to CD8 T-cell infiltration in this study was associated with their abnormally prolonged presence. Diet-induced obesity and insulin resistance, too, is associated with chronic inflammation in insulin-sensitive tissues (Khan et al., 2014, 2015). Therefore, it may be that the metabolic dysfunction caused by conventional T-cells in muscle and fat is due to the chronic inflammation associated with excessive feeding. Duchenne muscular dystrophy patients suffer from constant damage and inflammation in skeletal muscle, which could potentiate damage-worsening activity of the T-cells in the muscle. Also consistent with the idea that the persistent and unregulated presence of conventional T-cells can contribute to muscle pathology is that Tregs are beneficial for Duchenne muscular dystrophy and inflammatory myopathies (Waschbisch et al., 2010; Schenk et al., 2011; Villalta et al., 2014). Tregs have the capacity to suppress the activity of cytotoxic T-cells in inflammatory myopathies (Waschbisch et al., 2010), and attenuate inflammation in pathological conditions including Duchenne muscular dystrophy (Villalta et al., 2014). Thus, it seems that T-cells are beneficial for muscle regeneration in otherwise healthy muscle, so long as their presence and activity are properly regulated. The features of T-cell-deficient muscle repair and the known mechanisms by which T-cells support muscle repair are reviewed in the following section.

## How Do T-Cells Support Muscle Repair?

### Regulate Myogenic Cell Activity

T-cells might support muscle repair by regulating the activity and maintaining vitality of myogenic cells. T-cell-conditioned culture media promoted both migration (Dumke and Lees, 2011) and proliferation (Dumke and Lees, 2011; Fu et al., 2015) of muscle stem cells *in vitro*. Moreover, muscle stem cells cultured in T-cell-conditioned media for prolonged periods of time retained their ability to proliferate over many passages, and their myogenic potency (Fu et al., 2015). The researchers also identified an irreducible combination of 4 cytokines (IL-1α, TNF-α, IFN-γ, and IL-13) that accounted for the ability of the T-cell conditioned media to maintain muscle stem cell potency. While this study has important implications for muscle stem cell therapy, it also speaks to a potential mechanism of T-cell-mediated muscle regeneration; promoting a conducive microenvironment for effective satellite cell maintenance and function. However, because both of these studies (Dumke and Lees, 2011; Fu et al., 2015) used conditioned cell culture media from a general population of activated T-cells (CD3 positive), no conclusions can be made about the relative contributions of the specific subpopulations of T-cells on muscle stem cell activity and vitality.

A few studies have reported that Tregs influence the activities of muscle stem cells. Castiglioni et al. (2015) observed that T-cell deficient mice displayed impaired muscle regeneration and blunted satellite cell proliferation following injury. On the other hand, wild type mice had successful regeneration concomitant with T-cell recruitment and a robust increase in satellite cell number following injury. Because the T-cells that infiltrated the wild type muscle were particularly enriched with Tregs, the investigators asked whether the absence of Tregs in the knockout mice caused the deficiency in satellite cell expansion, which led to the reduced regeneration capacity. To test this, the researchers co-cultured satellite cells with either iTregs, or nTregs that were obtained from the spleen. iTregs (induced Tregs) are derived from naïve CD4 T-cells that were activated in a Treg-polarizing environment, while nTregs (natural Tregs) were committed to the Treg lineage from development in the thymus. Only iTregs were found to enhance satellite cell kinetics by facilitating proliferation. Thus, the researchers concluded that iTregs are important for muscle regeneration by promoting satellite cell proliferation. One criticism of this paper is that a previous study (Burzyn et al., 2013) had shown that the Tregs present in muscle following injury were predominantly nTregs, not iTregs. Moreover, data presented in the same paper showed that the “muscle Tregs” are of a highly specialized subset of nTregs that accumulate in damaged muscle with characteristic T-cell receptor arrangements and gene expression profiles (Burzyn et al., 2013). Thus, the method of co-culturing satellite cells in the presence of general Treg subsets may be too derived and non-specific for drawing strong conclusions.

Other studies (Burzyn et al., 2013; Kuswanto et al., 2016) have found that Tregs may be beneficial for muscle regeneration in part thanks to the secreted growth factor amphiregulin. The absence of Tregs following muscle damage resulted in altered whole muscle transcriptomic profiles, smaller nascent myofibers, and increased fibrosis. Treating Treg-less mice with amphiregulin normalized the muscle transcriptome and reduced expression of extracellular matrix-related genes. Treg-derived amphiregulin may exert some of its muscle regenerative functions by supporting myogenic differentiation as myoblasts treated with amphiregulin exhibited better myogenic potency *in vitro* (Burzyn et al., 2013). This suggests that Tregs may be promoting myogenic differentiation, not proliferation as was concluded by Castiglioni et al. (2015). Further evidence that Tregs might be impacting satellite cell function is that muscles enriched in Treg content following injury had a larger pool of satellite cells that were also better able to form myogenic colonies *ex vivo* (Kuswanto et al., 2016). These findings suggest that Tregs may be an important subset of T-cells that help maintain satellite cell stemness as demonstrated by Fu et al. (2015).

Whether Tregs support muscle regeneration directly by influencing satellite cells or indirectly, by regulating immune cell activity has yet to be definitively determined. Because of the well-established immunosuppressive functions of Tregs, it is likely that they affect muscle regeneration indirectly by regulating the activity of other lymphocytes and myeloid cells involved in muscle repair. A recent study showed that Tregs possessed immunosuppressive functions as well as direct amphiregulin-dependent tissue damage-protective function that was independent of immunosuppressive activity (Arpaia et al., 2015). This study was performed on lung tissue of mice with influenza infection using a Treg-specific knockout of amphiregulin. A similar study carried out in the context of muscle damage and regeneration would be insightful.

### Regulate Muscle Immune Cell Infiltrate

Effective muscle regeneration is dependent on the infiltration of inflammatory monocytes that differentiate into pro-inflammatory (M1) macrophages during the early stages of muscle repair and subsequently mature into anti-inflammatory macrophages (M2) during the later stages of muscle repair (Arnold et al., 2007, 2015; Ruffell et al., 2009; Tidball and Villalta, 2010; Lu et al., 2011). Recent studies show that a couple populations of T-cells are needed for both the recruitment of inflammatory monocytes, as well as governing their phenotypic evolution over the course of the muscle repair process.

Chemokines are small signaling molecules that are capable of directing the migration of immune cells. One particular chemokine, CCL2 (also known as MCP-1), appears to be especially important to recruit monocytes to injured muscle and is thereby important muscle healing process (Sun et al., 2009; Lu et al., 2011). CD8 positive T-cells facilitate the expression of CCL2 by resident macrophages in injured muscle (Zhang et al., 2014), and this interaction is crucial for the recruitment of inflammatory monocytes to the injured muscle. CD8 knockout mice failed to increase CCL2 expression and suffered from impaired muscle regeneration marked by blunted satellite cell expansion, increased fibrosis, and reduced cross-sectional area of regenerating myofibers.

**FIGURE 1:**
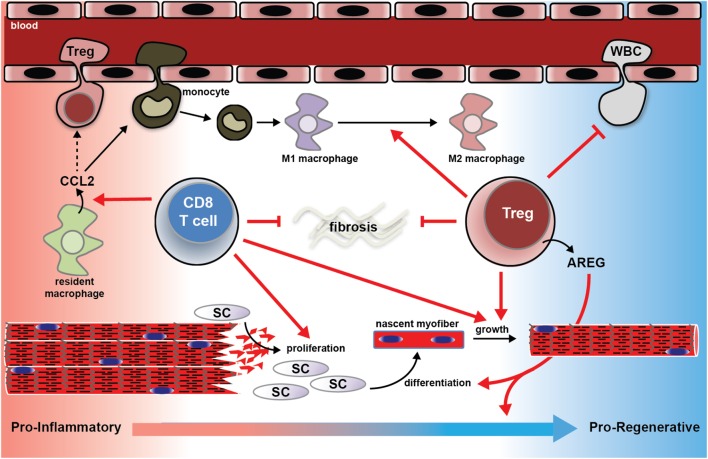
Schematic summary of the known mechanisms by which T-cells support muscle regeneration and recovery from traumatic injury. CD8+ T-cells facilitate CCL2 expression by muscle resident macrophages, which is essential for the recruitment of pro-inflammatory monocytes to the injured muscle. In the absence of CD8+ T-cells, pro-inflammatory monocyte recruitment is blunted, satellite cell pool is reduced, nascent myofiber growth is attenuated, and matrix deposition is exacerbated (see Zhang et al., 2014). Regulatory T (Treg) cells support muscle regeneration in part by the growth factor amphiregulin (AREG). Muscle Tregs express high levels of AREG. AREG treatment normalized the evolution of the muscle transcriptome over the course of the muscle repair process, and promotes myogenic differentiation *in vitro* (Burzyn et al., 2013). Muscle lacking or deficient in Tregs following injury suffer from exaggerated extracellular matrix deposition, slowed nascent fiber growth, and exaggerated inflammation, and failure of M1 macrophages to mature into M2 macrophages (Burzyn et al., 2013; Kuswanto et al., 2016). CCL2; C-C motif chemokine ligand 2; SC; satellite cell; WBC; white blood cell.

A recent study (Burzyn et al., 2013) found that in mice lacking Tregs, the recruitment of inflammatory monocytes to injured muscle was not diminished, but these cells failed to mature into M2 macrophages. Moreover, there was an overall greater influx of leukocytes into Treg-less muscle. Together these studies show that CD8 T-cells and Tregs work in concert to recruit monocytes to injured muscle, regulate their phenotype over the course of the regeneration process, as well as temper the overall inflammation. A schematic, illustrating the known contributions of T-cell activity to muscle repair is shown in **Figure 1**.

## What Signals Cause T-Cells to Accumulate in Damaged Muscle?

Given the importance of T-cells in the process of effective muscle regeneration, understanding the mechanisms of T-cell recruitment to injured muscle may harbor some therapeutic applications. However, little information is available on this topic. One study showed that CCL5 secreted by intramuscular macrophages was instrumental in recruiting CD8 T-cells to damaged muscle (Kohno et al., 2011). However, this study used a knockout mouse that suffered from exaggerated and prolonged T-cell accumulation following injury. Moreover, a CCL5-neutralizing antibody successfully blunted T-cell accumulation only in the knockout animal – the same treatment did not reduce T-cell recruitment in the wild type animals. Thus, it seems doubtful that CCL5 is involved in T-cell recruitment to healthy muscle. Recently, IL-33 was found to be important for the accumulation of Tregs in injured muscle, yet IL-33 did not cause T-cell chemotaxis. Instead, IL-33 enriched the muscle Treg content by promoting proliferation and reducing lymphatic egress (Kuswanto et al., 2016). Another study from the same lab found that muscle Tregs express CCR2 (the receptor for CCL2) to a much greater degree than Tregs found in the spleen (Burzyn et al., 2013). This suggests that Tregs may be drawn to injured muscle by CCL2, just as monocytes are. If indeed the CCL2/CCR2 axis is needed for T-cell recruitment to damaged muscle, the literature regarding CCL2 or CCR2 in muscle regeneration might need to be reevaluated. This is because monocytes/macrophages were thought to be the main cell types impacted by the disruption of this signaling axis (Contreras-Shannon et al., 2007; Shireman et al., 2007; Sun et al., 2009; Lu et al., 2011; Arnold et al., 2015). In a study done in humans, muscle CD8 T-cell content following contraction-induced injury was correlated with the muscle CXCL10 content (Deyhle et al., 2015). The CXCL10/CXCR3 axis is an important peripheral homing system for T-cells in many immunological contexts (Groom and Luster, 2011). Therefore, these data (Deyhle et al., 2015) suggest CXCL10 may be important for T-cell recruitment to damaged muscle, yet that question remains to be investigated.

## Do T-Cells Respond to Contraction-Induced Muscle Damage?

Exercise-induced, or contraction-induced muscle damage is usually mild compared to other injuries with a more traumatic cause. There is little information on whether T-cells are involved in repair and adaptation to contraction-induced damage. An early investigation (Malm et al., 2000) cast doubt on this idea. In this study, it was concluded that the degree of T-cell infiltration following eccentric cycling was too small to be practically significant (although CD8 and CD4 T-cells were elevated in the muscle post-damage). More recently, a report was published on a group of trained ultra-endurance athletes who completed a 24-h-long bout of exercise (Marklund et al., 2013). This activity resulted in substantial muscle damage. One day after the exercise bout there was a significant increase in the number of CD3 positive T-cells in the muscle. Among those T-cells, most were CD8 positive. In addition, there was a significant increase in major histocompatibility complex class I (MHC I) present on the muscle fibers. MHC I presents peptides on the cell surface for CD8 T-cell recognition. Because healthy and unperturbed skeletal muscle normally expresses low levels of MHC I (Wiendl et al., 2005; Graca and Kouyoumdjian, 2015), these data suggest that upon contraction-induced injury, skeletal muscle may up regulate MHC I and communicate with the CD8 T-cells. The function of these T-cells and whether they were detrimental, beneficial or passive observers to the muscle damage/repair process following contraction-induced damage is not known. We recently published a report (Deyhle et al., 2015) also showing that CD8 T-cells were increased in muscle following contraction-induced injury. However, we used a less extreme stimulus consisting of high force lengthening contractions. In contrast to Marklund et al. (2013), we found no significant increase in MHC I following damage. However, this does not rule out the possibility of important muscle-to-T-cell communication; the quality (the specific peptide sequence), not the quantity (overall amount) of the presented peptide is paramount to T-cell activation (Klein et al., 2014). Though no definitive conclusions as to the functional significance of these cells can be drawn from this study, we did find histological evidence of T-cells infiltrating damaged and regenerating muscle fibers. Another interesting observation from this study was a higher number of CD8 T-cells was present in the muscle following a second bout of lengthening contractions, when the muscle was adapted to the stimulus. Because muscle that was adapted to lengthening contractions (and therefore resistant to damage) had more T-cell accumulation than non-adapted muscle, it appears less likely that they are playing a detrimental role. Together these data support that T-cells do indeed respond to contraction-induced damage. A notable deficiency in the available literature is that studies thus far have only used immunoshistochemistry to assay for T-cells in the muscle after contraction-induced damage (Malm et al., 2000; Marklund et al., 2013; Deyhle et al., 2015). A multicolor flow cytometric assay of muscle T-cells following contraction-induced damage is needed to get a more detailed picture of the T-cell subsets that respond to this type of injury.

Compared to studies investigating intramuscular T-cells, more research has investigated the effect of exhaustive or prolonged exercise on circulating T-cell numbers and phenotype (Clifford et al., 2017; Shaw et al., 2018). The aims of such investigations are primarily to understand the immunosuppressive effects of exhaustive exercise, however, whether these changes in circulating T-cells influence muscle repair and regeneration remains to be determined.

More study is needed to determine if T-cells play as important a role in muscle repair from contraction-induced injury as they do in repair from traumatic muscle injury. In addition to their potential role in muscle repair, the presence of T-cells in the muscle following muscle-damaging exercise highlights the possibility of a role in realizing exercise adaptations, such as muscle hypertrophy and damage resistance (the repeated bout effect) (Hyldahl et al., 2017). Contraction-induced damage leads to an increase in muscle satellite cell content (Hyldahl et al., 2014). As reviewed above, T-cells appear to be necessary for effective satellite cell proliferation and function following a traumatic injury (Burzyn et al., 2013; Zhang et al., 2014; Castiglioni et al., 2015; Fu et al., 2015). Thus, it is possible that T-cells may be important to promote muscle repair and adaptation following contraction-induced injury by promoting/regulating satellite cell activity. A loss of function approach, whereby T-cell subsets are punctually deleted in the face of muscle-damaging exercise would help answer these remaining questions.

## A Muscle Damage Memory Mechanism?

The observation that T-cells accumulate in greater numbers following a repeated bout of damaging contractions, when recovery is accelerated [the repeated bout effect (Deyhle et al., 2015; Hyldahl et al., 2017)], suggests that T-cells may be facilitating repair by employing their defining immunological attribute: memory. It may be that muscle damage generates muscle damage-specific peptides that are loaded and presented on MHC for T-cell recognition, resulting in their activation and recruitment to damaged muscle where they would carry out muscle repairing and damage-protective effector functions. At the conclusion of the muscle repair, the population of participating T-cells would be expected to contract via apoptosis, but not without leaving behind surviving memory cells. These damage-experienced memory T-cells would be poised for more rapid expansion and muscle repairing functions when re-exposed to their conjugate damage-specific peptides upon a repeated bout. Thus, just as T-cells develop memory of specific microbial peptides affording more effective and rapid immune responses, so too they may develop memory of damage-specific peptides and more effectively facilitate muscle repair, thereby mediating the repeated bout effect. The most intriguing data consistent with this hypothesis is from a study (Burzyn et al., 2013) that sequenced the complementary determining region 3 (that determines peptide specificity) of the α and β T-cell receptor genes from individually sorted T-cells harvested from regenerating muscle. Strikingly, an identical T-cell receptor arrangement was found repeatedly among different animals in separate experiments. This suggests that just as T-cells develop memory of peptides derived from infectious pathogens, they might also generate memory of muscle damage-specific peptides, resulting in resistance to future muscle damage. The possibility of this phenomenon has been noted in another recent review (Tidball, 2017). However, this hypothesis awaits the verification of future experiments. Adoptive T-cell transfer experiments could be useful to test if T-cells can confer muscle damage-protective adaptation of the repeated bout effect.

## Conclusion

T-cells are capable of contributing to the pathology of many muscle diseases. Recently, studies have shown that T-cells are also necessary participants in successful muscle repair/regeneration following injury. The mechanisms by which T-cells aid in muscle repair are yet to be firmly established, but likely include nurturing/regulating muscle stem cell activity and regulating the activity of other leukocytes during the inflammatory and regenerative phases after injury. A few studies have shown that T-cells also accumulate in muscle following damaging muscle contractions. More investigation is needed to determine the necessity of T-cells in muscle repair from and adaptation to damaging muscle contractions.

## Author Contributions

MD wrote and edited this mini review. RH helped write and edit this mini review.

## Conflict of Interest Statement

The authors declare that the research was conducted in the absence of any commercial or financial relationships that could be construed as a potential conflict of interest.
